# Research progress on risk factors related to intracranial artery, carotid artery, and coronary artery stenosis

**DOI:** 10.3389/fcvm.2022.970476

**Published:** 2022-10-25

**Authors:** Ruijun Liu, Jing Shao

**Affiliations:** ^1^Department of Neurointerventional, The Third Hospital of Jinan, Jinan, China; ^2^Department of Cardiovascular, The Third Hospital of Jinan, Jinan, China

**Keywords:** risk factors, intracranial artery stenosis, carotid artery stenosis, coronary artery stenosis, atherosclerosis

## Abstract

In recent decades, with the rapid development of economy, the acceleration of social aging and urbanization, and the prevalence of unhealthy lifestyles, the number of patients with cardiovascular and cerebrovascular diseases has shown an increasing trend year by year. It has also become one of the important causes of disability and death in all ages and groups. Atherosclerosis is the main pathological change of ischemic cardiovascular and cerebrovascular diseases, which mainly invades the large and medium arteries of the body circulation. In particular, cerebral artery and coronary artery lesions have the most significant impact on life. There is the same pathogenic mechanism between intracranial and extracranial arteries and coronary atherosclerosis, so there is a certain relationship between the degree of atherosclerosis. In this paper, the risk factors related to intracranial and extracranial arteries and coronary artery stenosis were reviewed. It provides a theoretical basis for early detection, early diagnosis and early treatment of intracranial and extracranial artery and coronary artery stenosis to reduce the occurrence and development of cardiovascular and cerebrovascular diseases.

## Introduction

The relationship between intracranial artery, carotid artery and coronary artery atherosclerosis: there is the same pathogenic mechanism between intracranial artery, carotid artery and coronary artery atherosclerosis, so there is a certain relationship between the degree of atherosclerosis. Zhou (1) found that carotid artery is closely related to the degree of coronary heart disease and cerebrovascular and coronary artery have consistency in the increase of vascular stenosis. However, the study of Guo et al. (2) showed that there was no significant correlation between intracranial and extracranial artery stenosis and asymptomatic coronary artery stenosis. The incidence of coronary artery stenosis and multi-vessel stenosis would also gradually increase with the deepening of extracranial artery stenosis, but there was no statistical difference between them. Studies suggest that atherosclerosis first occurs from large elastic aorta, and then gradually involves small arteries (such as limb arteries, coronary arteries, intracranial arteries). Carotid atherosclerosis and main atherosclerosis occur at approximately the same time, earlier than coronary atherosclerosis. It has been reported in the literature that the occurrence time of arterial lesions in carotid artery and coronary artery is roughly the same, even slightly earlier than that in coronary artery (3, 4). However, studies by Guo et al. (5) have shown that the detection rate of coronary artery stenosis is higher than that of carotid plaque, suggesting that coronary artery stenosis occurs earlier than CAS. The reasons may be as follows: (1) The diameter of coronary artery is smaller than that of carotid artery, which is easy to cause stenosis; (2) The coronary artery on the surface of the myocardium is susceptible to myocardial pulsation, and is pulled by the myocardium to cause damage to the wall and even stenosis. Some domestic studies have also confirmed that extracranial artery is prone to stenosis with intracranial artery stenosis, and the latter occurs earlier than the former, suggesting that extracranial artery stenosis predicts more serious artery stenosis than intracranial artery stenosis. Therefore, the study of Guo et al. (5) showed that the degree of CAS stenosis was more accurate in indicating coronary heart disease patients with relatively severe coronary stenosis. The distribution of intracranial and extracranial artery stenosis is different. Lin et al. (6) have shown that patients with large artery atherosclerotic stenosis in Western countries are more likely to have carotid artery stenosis, while patients in Asian countries are more likely to have intracranial artery stenosis. Song et al. (7) showed that coronary heart disease complicated with intracranial artery stenosis mainly involved ICA system, MCA stenosis was the main cause of ICA system, and internal carotid artery and vertebrobasilar artery system showed an increasing trend of vascular stenosis with the expansion of coronary artery stenosis. According to the statistics of 6,352 autopsy cases in China, the left anterior descending branch was the most common site of coronary stenosis, followed by the right trunk and the left circumflex branch. There are certain differences in the stenosis of intracranial and extracranial arteries and coronary arteries. Zhang et al. (8) have shown that patients with coronary artery and intracranial artery stenosis are more complicated with carotid atherosclerotic plaques, and the nature of plaques is multiple and heterogeneous, indicating that carotid atherosclerotic plaques are multi-site lesions, unstable and easy to fall off. At the same time, the instability of carotid artery plaque also indicates the instability of coronary artery plaque. The number and nature of carotid artery plaque in patients with coronary heart disease have a good predictive effect on the diagnosis of ischemic cerebrovascular disease.

The possible mechanism of carotid atherosclerosis leading to cerebral infarction (9): (1) the carotid artery is affected by blood flow shock for a long time, and its plaque is easy to damage, unstable and fall off. The small plaque that falls off after rupture will block the distal vessel with blood flow operation. (2) the carotid atherosclerotic plaque is not smooth, and it is easy to aggregate platelets and coagulation factors, leading to thrombosis and vascular stenosis. In this process, it may also be involved in the low perfusion based on carotid stenosis. When the degree of carotid artery disease is mild, the body can maintain relatively stable cerebral blood flow through self-regulation mechanisms such as collateral circulation or distal vascular dilatation, thereby reducing peripheral vascular resistance. With the aggravation of the lesion degree and the decrease of the peripheral perfusion pressure, it causes decompensation and eventually leads to the decrease of the peripheral perfusion pressure, leading to the occurrence of cerebral infarction. Liu et al. (10) have found that extracranial carotid artery stenosis is closely related to the incidence of cerebral infarction, and the risk of cerebral infarction is closely related to the degree of extracranial carotid artery lesions, with the highest risk of vertebral artery lesions, followed by the beginning of the internal carotid artery. At the same time, studies have also shown that patients with cerebral infarction lesions on the same side of the extracranial carotid artery than the contralateral extracranial carotid artery vascular lesions. Other studies in China have found that extracranial carotid stenosis in patients with cerebral infarction is the most common in the carotid artery enlargement and the beginning of the internal carotid artery, which may be closely related to the fact that this part is a common site for carotid plaque. As for the specific reasons, some studies have found that the slow blood flow in this part may easily lead to the erection of blood lipids, destroy the arterial intima and vascular wall, and lay the foundation for the occurrence of plaque and the formation of stenosis (11).

**Figure 1:**
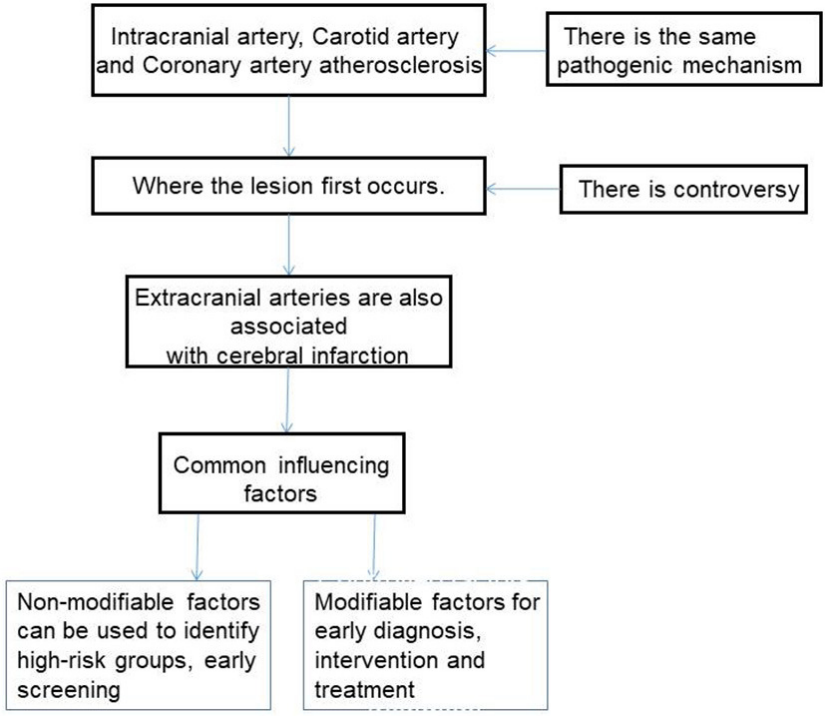
The narrative idea of this article.

In clinical research, it is found that cardiovascular and cerebrovascular diseases occur mostly in the middle-aged and elderly population, which not only has a high incidence, but also shows a trend of younger age. In addition, the onset is relatively acute and the disease progresses rapidly, which has a serious impact on the quality of life and health of patients, and even endangers life (12, 13). Therefore, early detection, early diagnosis and early treatment of intracranial and extracranial artery and coronary artery stenosis and prevention of restenosis are of great significance to reduce the occurrence and development of cardiovascular and cerebrovascular diseases. This article briefly discusses the risk factors of intracranial and extracranial artery and coronary artery stenosis (Figure 1).

## Risk factors for intracranial and extracranial artery and coronary stenosis

Intracranial artery, carotid artery and coronary artery stenosis are the most common clinical manifestations, which mainly lead to heart and cerebral ischemia, severe symptoms, difficult recovery and even serious sequelae, increasing the family, and social burden of patients. Therefore, it is crucial to avoid premature stenosis or slow down the progress of stenosis in intracranial and extracranial and coronary arteries, and timely intervention and treatment. Therefore, we need to pay attention to and prevent the risk factors leading to intracranial artery, carotid artery and coronary artery stenosis, and then implement individualized early intervention and secondary prevention. With the increasing prevalence of cardiovascular and cerebrovascular diseases caused by intracranial and extracranial artery and coronary artery stenosis, a large number of literatures on the risk factors of its stenosis have emerged. There are many risk factors related to its stenosis, which are roughly divided into two categories, namely modifiable and non-modifiable risk factors (Table 1). Non-modifiablerisk factors include age, gender, race, family history, etc. The modifiable risk factors include hypertension, diabetes, dyslipidemia, carotid intima-media thickness, hyperhomocysteinemia, high lipoprotein (a), smoking history, long-term alcohol history, obesity/overweight, hyperuricemia, high-sensitivity C-reactive protein.

**Table 1:** Correlative risk factors of intracranial artery, carotid artery, and coronary artery stenosis.

**Factor category**	**Factors**	**References**	**Summarize**
Non-modifiable risk factors	Age	Verheggen et al. (14) Jiang et al. (15) Wang (16) Ouchi et al. (17) Kwon et al. (18)	The older the age, the higher the risk of intracranial artery stenosis, the greater the degree of stenosis
	Gender	Tian et al. (19) Pluta et al. (20) Jin et al. (21) Zhao et al. (22) Segura et al. (23) Turan et al. (24) Wong et al. (25)	The incidence of ischemic stroke is greatly affected by gender differences, and the incidence in males is generally higher than that in females
	Race	Kim et al. (26) Wong et al. (27) Huang et al. (28) Saunders et al. (29) Xu et al. (30)	The location of vascular stenosis is also different due to race. Different people had different coronary atherosclerosis and AS outcomes. Black patients with coronary heart disease are more likely to evolve into myocardial infarction and heart failure.
	Family History	Morrison et al. (31) Lu et al. (32) Hao et al. (33) Wang et al. (34) Seshadri et al. (35) Chung et al. (36) Li et al. (37) Fisher et al. (38) Koleganova et al. (39) Ciccone et al. (40) Gao et al. (41)	Family history not only affects the occurrence of ischemic cerebrovascular disease, but also has a certain role in the occurrence and development of coronary heart disease. People with family history of coronary heart disease have a higher probability of acute myocardial infarction
Modifiable risk factors	Hypertension	Wang et al. (42) Kjeldsen et al. (43) Liu et al. (44) Thomas et al. (45) Cao et al. (46) Xiao Youyuan. (47) Yang et al. (48) Liu et al. (49)	Hypertension was most closely related to intracranial artery stenosis, especially in patients with coronary artery stenosis. Hypertension can damage the intracranial artery through a variety of mechanisms, and can directly damage the small arteries with a diameter of 50 ~ 200 um, resulting in intimal ischemic damage, increased permeability and vitreous degeneration.
	Type 2 diabetes	Lee et al. (50) Zhang et al. (51) Inzucchi et al. (52) Selvin et al. (53) Weber et al. (54) Subramanian et al. (55) Wang et al. (56) Liu et al. (57)	The effect of diabetes on vascular system is systemic, regardless of the size of blood vessels, are damaged by diabetes. Studies have shown that diabetes not only damage the intracranial artery, but also damage the extracranial artery, diabetes is an independent risk factor for intracranial artery and coronary artery stenosis has been confirmed by many clinical trials at home and abroad
	Abnormal lipid metabolism	Thomas et al. (45) Wong et al. (58) Turan et al. (24)	Dyslipidemia is an independent risk factor for intracranial and extracranial artery and coronary artery stenosis. Hypercholesterolemia was associated with intracranial artery stenosis, especially with middle cerebral artery.
		Yasaka et al. (59) Shen et al. (60) Zhu et al. (61) Gotto et al. (62) Park et al. (63)	
	Carotid artery intima-media thickness (IMT)	Wang et al. (56)	Because IMT is the degree of AS lesions directly seen and is the real manifestation of arterial lesions that have occurred, and hypertension is affected by multiple factors, whether it must cause ICAD and CHD diseases needs to be further confirmed.
	Hyperhomocysteinemia	Wang et al. (64) Neetu et al. (65) Eleftheriadou et al. (66) Temple et al. (67) Liu et al. (68) Xiong et al. (69)	Hyperhomocysteine was not only positively correlated with intracranial artery stenosis, but also had some relationship with coronary artery stenosis.
	High lipoprotein (a)	Erqou et al. (70) Calmarza et al. (71) Huang et al. (72)	LP (a) is recognized as a risk factor for coronary heart disease and stroke. Studies have shown that there are lipoprotein (a) in serum and AS plaque, women are higher than men, plaque concentration is higher than serum, unstable plaque is higher than stable plaque. Serum Lp (a) content is closely related to the lesion range of coronary artery stenosis, and the number of diseased coronary arteries increases with the increase of Lp (a) content.
	History of smoking	Wang et al. (73) Cheng (74) Kim et al. (75) Centers for Disease Control and Prevention (76)	Smoking is an independent risk factor for intracranial artery stenosis Smoking was closely related to the aggravation of AS, which could increase the progression rate of AS by 50%, especially for patients with hypertension. The probability of AS in smokers and non-smokers is significantly different. After eliminating the influence of potential risk factors, smoking is closely related to the incidence of intracranial artery stenosis.
	Long-term history of alcoholism	Melgaard et al. (77) Gdovinová (78)	Most studies have shown a J-shaped curve between alcohol intake and the risk of cerebrovascular disease, that is, mild drinking may have a protective effect on cerebrovascular disease, but moderate or even excessive drinking can lead to a sharp increase in the prevalence of cerebrovascular disease.
	Obesity/overweight	Zhang et al. (79) Milionis et al. (80) Song et al. (81) Irace et al. (82)	The increase of BMI is closely related to cellular inflammatory response, abnormal blood lipid metabolism, increase of oxidative free radicals, increased platelet aggregation ability, increased coagulation function, and decreased activity of the system in the state of insulin resistance and insulin resistance, leading to atherosclerosis and plaque formation under the interaction of various factors.
	Hyperuricemia	Zhang et al. (83) Kim et al. (84) Okura et al. (85)	Hyperuricemia (HUA) is one of the risk factors of intracranial artery stenosis. The content of serum uric acid was significantly increased in patients with severe intracranial artery stenosis, suggesting that the content of uric acid is closely related to the degree of intracranial artery disease.
	High-sensitivity C-reactive protein	Yu et al. (86) Ding et al. (87) Chen et al. (88)	The level of Hs-CRP in serum was related to the echo intensity and surface morphology of atherosclerotic plaques. The degree of coronary stenosis in CHD patients is significantly correlated with the level of Hs-CRP, which can be used to determine the degree of coronary lesions.
	Lifestyle behaviors	Scicchitano et al. (89) Matey-Hernandez et al. (90) Kavey et al. (91) Pauwels (92) Jeong et al. (93)	Individuals with cardiovascular disease may benefit from physical activity to a greater extent than do healthy subjects without cardiovascular disease. Lifestyle behaviors including diet, physical activity and sedentary behavior are key modifiable risk factors for many diseases and improving these behaviors is considered essential to reducing the financial and health burden of diseases.

## Non-modifiable risk factors

### Age

Age is a non-modifiable independent risk factor for coronary heart disease and intracranial artery stenosis. Previous studies have shown that the incidence of ischemic cerebrovascular disease and the risk of cerebrovascular stenosis will continue to increase with age. When the age increases by 10 years after 55 years old, the risk of cerebrovascular disease is twice the original (14). Studies have confirmed that the number of extracranial arterial lesions is positively correlated with age. For every 1-year-old increase in age, the possibility of abnormal extracranial artery stenosis is 1.070 times that of the original (15). The incidence of extracranial artery stenosis increases with age, especially in the elderly. Wang (16) found that age was an important risk factor for intracranial artery stenosis. The incidence of intracranial and extracranial vascular stenosis is roughly equal when the age is about 70 years old. The incidence of intracranial vascular stenosis is higher than that of extracranial vascular stenosis when the age is less than 70 years old, and the incidence of extracranial vascular stenosis is higher than that of intracranial vascular stenosis when the age is more than 70 years old. Ouchi et al. (17) reported that the incidence of asymptomatic cerebral infarction increased significantly with age. Kwon et al. (18) reported that the older the age, the higher the risk of intracranial artery stenosis, the greater the degree of stenosis.

### Gender

Gender is a non-modifiable risk factor, and many previous studies have shown that it is one of the risk factors for ischemic stroke (19). Epidemiological surveys at home and abroad have found that the incidence of ischemic stroke is greatly affected by gender differences, and the incidence in males is generally higher than that in females, with a ratio of ~1.1~1.5:1 (20– 22); meanwhile, previous studies have also demonstrated that arterial stenosis in males is more common in extracranial segmental arteries, while intracranial arterial stenosis in females (23–25).

### Race

Intracranial artery, carotid artery and coronary artery stenosis are the common causes of ischemic cardio-cerebral vascular disease. Studies have shown that for stroke patients, the genetic susceptibility of intracranial and extracranial artery stenosis varies significantly with race. At the same time, the location of vascular stenosis is also different due to race. For example, extracranial carotid artery lesions are more common in white people, especially in European and American populations, while Spanish and Asian people are more likely to cause diseases due to intracranial artery lesions, especially in the middle cerebral artery (26). There are some differences between different races. Chinese people have higher incidence of intracranial artery stenosis than white people. Unlike white people who are prone to extracranial artery stenosis, ischemic stroke in Asians and black people is mostly caused by intracranial artery lesions (27, 28). Epidemiological investigation also shows that about 8% of cerebral infarction in European and American whites is caused by intracranial artery stenosis, while as high as 33% of cerebral infarction patients in China have different degrees of intracranial artery stenosis (25). Moreover, the incidence of CHD varies greatly among different races. For example, compared with black people, white people have a significantly lower probability of CHD (29). Similarly, the risk of ICAD in white people is much lower than that in black people, and the probability of ICAD in white people is 2–4 times that in white people. In a comparative study of Asian people and white people (30), it was found that there were also differences in the location and severity of coronary atherosclerosis. The main vessels involved in atherosclerosis in Asian people and white people were left anterior descending branch, right coronary artery and left main coronary artery. The lesions of three branches and left main coronary artery in white people were 31.9 and 13.5%, and those in Asian people were 26.0 and 8.3%, respectively. The incidence of coronary porridge in white people was higher than that in Asian people, and the dominant type of right coronary artery in Asian people was more obvious. Different people had different coronary atherosclerosis and AS outcomes. Black patients with coronary heart disease are more likely to evolve into myocardial infarction and heart failure.

### Family history

In recent years, some researchers have found that family history plays a certain role in the occurrence and development of cerebrovascular diseases, and the reason may be related to genetic susceptibility and/or common living environment (31). Previous studies have confirmed that genetic factors play an important role in the pathogenesis of ICAD, and the occurrence of ICAD is also affected by the postnatal environment and the interaction between the two (32–34). The probability of ICAD in the population without family history is low (35), only 1/3–1/2 of the incidence in the population with family history. Studies have also found that the incidence of stroke recurrence in patients with family history is much higher than that in patients without family history (36). Li et al. (37) showed that in patients with acute cerebral infarction, patients with family history were prone to more serious neurological deficits, and the recovery was slow and the prognosis was poor. In addition, the “fetal programming” during critical phases of development in utero could be responsible for metabolic disturbances in adult life and long-term structural changes of the vascular system (38, 39). The study of Ciccone et al. (40) have shown that neonatal and maternal characteristics could early influence atherosclerosis development. Moreover, Family history not only affects the occurrence of ischemic cerebrovascular disease, but also has a certain role in the occurrence and development of coronary heart disease. Epidemiological survey confirmed that people with family history of coronary heart disease have a higher probability of acute myocardial infarction (41).

## Modifiable risk factors

### Hypertension

There are many studies on the impact of hypertension on AS and ICAD. Domestic literature reported that 19.8% of acute coronary heart disease and 41% of acute ischemic stroke events can be attributed to hypertension (42). Many studies have confirmed that hypertension is a powerful independent predictor of ICAD and CHD, and the incidence of hypertension in Chinese is higher than that in foreign countries, but the effect of blood pressure on the two is different. Domestic and foreign studies have shown that ICAD is more affected by hypertension than CHD. Kjeldsen et al. (43) confirmed that for CHD, the relationship curve between ICAD and systolic blood pressure was more steep, suggesting that hypertension was more closely related to ICAD than CHD. Zhang et al. (8) showed that hypertension was most closely related to intracranial artery stenosis, especially in patients with coronary artery stenosis. Hypertension leads to intracranial large artery atherosclerosis and leads to large artery stenosis or occlusion. In Chinese patients, intracranial artery stenosis is more common than extracranial artery stenosis, and MCA stenosis is the most common. It is not related to age, gender and stroke type. Hypertension is positively correlated with MCA stenosis (44), especially when systolic blood pressure is in a high state for a long time (45). Long-term high blood flow pressure continued and mechanically damaged endothelial cells, and the latter released inflammatory mediators to promote the proliferation of smooth muscle cells and aggravate their fibrosis, resulting in vascular wall thickening and remodeling, resulting in reduced vascular compliance. Arterial spasm will eventually lead to limited systolic and diastolic functions, and the damage to systolic function is more obvious, which seriously affects the blood flow velocity and increases vascular resistance (46). Studies have shown that the higher the blood pressure, carotid intima-media thickening is more obvious, the higher the incidence of plaque (47), easy to fall off with blood flow to block blood vessels to form ischemic cerebrovascular disease. More and more evidence shows that ICAD is not only closely related to systolic blood pressure, but also increases significantly with the increase of diastolic blood pressure and mean arterial pressure (48). With the increase of age, arterial elasticity gradually decreases, which leads to continuous increase of systolic blood pressure and continuous damage of arterial intima (49). Hypertension can damage the intracranial artery through a variety of mechanisms, and can directly damage the small arteries with a diameter of 50~200 um, resulting in intimal ischemic damage, increased permeability and vitreous degeneration.

### Type 2 diabetes

The effect of diabetes on vascular system is systemic, regardless of the size of blood vessels, are damaged by diabetes. Studies have shown that diabetes not only damage the intracranial artery, but also damage the extracranial artery (50), diabetes is an independent risk factor for intracranial artery and coronary artery stenosis has been confirmed by many clinical trials at home and abroad (51), including fasting plasma glucose (FPG), postprandial blood glucose (PPG) and glycosylated hemoglobin (HbA1c) are increased risk factors for intracranial artery stenosis, especially MCA stenosis. Studies have also shown that insulin resistance can also be used as an independent risk factor for intracranial artery stenosis (52). More and more studies have confirmed that the incidence of ICAD is significantly increased when the fasting blood glucose is greater than 5.5 mmol/L. The incidence of ICAD increases by 17% for every 1% increase in the level of glycosylated hemoglobin (HbA1c). The mechanism may be related to the damage of vascular wall caused by oxygen free radicals generated by the oxidation of blood glucose (53). Liu et al. (54) showed that diabetes was closely related to the severity of intracranial artery lesions. The higher the stenosis was, the higher the incidence of ICAD was and the worse the prognosis was. Weber et al. (55) found that diabetes was closely associated with ICAD recurrence in patients with intracranial vascular stenosis. Subramanian et al. (56) confirmed that diabetic patients with cerebral infarction were more likely to occur in the posterior circulation than in the anterior circulation through the study of 8,489 ICAD patients in Canada. Diabetic patients often have hypertension leading to arterial stenosis, but unlike hypertension, the incidence of diabetes in China is lower than that in foreign countries, while the incidence of hypertension in foreign countries is lower than that in China. Although both of them are closely related to the incidence of CHD and ICAD, some studies have proved that hypertension and diabetes have different effects on cardiovascular and cerebrovascular diseases. Hypertension has a significant effect on ischemic stroke than on coronary heart disease, while diabetes has a more significant effect on coronary heart disease. Studies have confirmed that in the complications of diabetic patients in China, stroke increases by 10 times, coronary heart disease increases by 24 times, and it is slightly lower in foreign countries, which are three times (57). The mechanism of diabetes-induced arterial stenosis is not very clear. Previous studies have shown that the possible mechanism is that hyperglycemia activates protein kinase C through the synthesis of diacylglycerol, which causes the thickening of the basement membrane of the wall and the weakening of the vasomotor function. At the same time, the persistent increase of blood glucose damages vascular endothelial cells through inflammatory factors, resulting in functional disorder. In the long run, it causes damage to the integrity of vascular structure and function, thereby promoting the occurrence of AS. This process requires the participation of inflammatory factors such as high-sensitivity C-reactive protein and tumor necrosis factor a. At the same time, the vascular injury caused by diabetes is also related to hyperinsulinemia, insulin resistance, dyslipidemia, vascular endothelial cell damage, enhanced platelet aggregation, platelet adhesion to vascular wall, and inhibition of fibrinolytic system activity (54).

### Abnormal lipid metabolism

Dyslipidemia is an independent risk factor for intracranial and extracranial artery and coronary artery stenosis, which has been proved by many studies (45, 58), including the increase of TC, TG, LDL-C and the decrease of HDL-C. Compared with gender, age, hypertension, diabetes and other risk factors, dyslipidemia is more prominent, which leads to intracranial artery stenosis. The reason is that any abnormality of TC, TG, HDL-C and LDL-C is judged as dyslipidemia. Turan et al. (24) found that dyslipidemia was significantly associated with intracranial artery lesions. Yasaka et al. (59) confirmed that hypercholesterolemia was associated with intracranial artery stenosis, especially with middle cerebral artery. Previous studies have shown that hypercholesterolemia is closely related to intracranial artery stenosis. A large number of studies have shown that the increase of LDL-C is an independent risk factor for AS. A study by Shen et al. (60) showed that the LDL level in the intracranial artery stenosis or occlusion group was higher than that in the non-intracranial artery stenosis group, and the difference was significant. LDL-C destroys the vascular endothelium, changes its permeability, and is oxidized to form low-density lipoprotein (ox-LDL), which stimulates smooth muscle cell proliferation and fibrosis, and may also be related to changes in the conformation of poB 100 in the LDL molecule, which reduces the recognition of LDL-C by the LDL receptor and increases the interaction with scavenger receptors, which is conducive to the formation of foam cells and the occurrence of AS, ultimately causing the occurrence of vascular stenosis (61). The role of HDL is to transport cholesterol in surrounding tissues and organs such as the vessel wall to the liver tissue for decomposition and metabolism, reducing the deposition of cholesterol in the vessel wall, so HDL is a protective factor for atherosclerosis (62). In addition, Park et al. (63) concluded that Apo-A and Apo-B are closely related to the degree of intracranial artery disease. Apo-B is the main component of protein in LDL-C particles, and its increased level can promote the formation of AS. Apo-A and Apo-B ratio are significantly correlated with the degree of intracranial artery stenosis.

### Carotid artery IMT

Atherosclerosis is a systemic lesion that mainly invades large and medium-sized arteries, with simultaneous involvement of both coronary and carotid arteries rare. In the early stage of AS, the arterial intima is mostly injured, with arterial intimal thickening as the main manifestation, while arterial stenosis is a more serious manifestation of AS, mostly present in the late stage of AS. An increasing number of studies have suggested a tight link between IMT and coronary artery stenosis and have been used as the most relevant predictor of coronary artery stenosis. The incidence of acute myocardial infarction is significantly increased in patients with carotid atherosclerosis, with an 11% increased risk of adverse cardiovascular events per 0.1 mm of carotid intima-media thickness. At the same time, its thickness also has a certain relationship with the stenosis of intracranial vessels. IMT is one of the causes of ICAD. The detection rate of intracranial artery stenosis increases with the degree of coronary artery and carotid artery stenosis. It has been reported in the domestic literature that IMT has a higher predictive ability for cardiovascular and cerebrovascular diseases than other risk factors, and IMT has a suggestive significance for intracranial vascular stenosis compared with hypertension. Because IMT is the degree of AS lesions directly seen and is the real manifestation of arterial lesions that have occurred, and hypertension is affected by multiple factors, whether it must cause ICAD and CHD diseases needs to be further confirmed (57).

### Hyperhomocysteinemia

In recent years, the effect of hyperhomocysteinemia on cerebral vessels has attracted more and more attention. The research on the increase of Hcy has become one of the hotspots in basic and clinical medical research. There are also many controversies about the research conclusion of hyperhomocysteinemia on the degree of arterial stenosis. Wang et al. (64) showed that Hcy was related to the lesion range and degree of AS. Homocysteine (Hcy) is an important intermediate substance generated during methionine metabolism, which can damage the vascular wall and is closely related to the damage of all major arteries in the body. The mechanism of cerebral vascular stenosis caused by high Hcy is not very clear. At present, some researchers believe that the toxic effect of high Hcy: one is to damage the vascular endothelial (65); second, elevated Hcy causes vascular wall inflammation. The above two reasons aggravate the damage of vascular wall and promote the formation of AS, and interfere with normal physiological metabolism such as sugar, fat and protein, resulting in arterial stenosis caused by lipid deposition. The study by Eleftheriadou et al. (66) showed: when Hcy levels were> 14 umo1/L, the risk of senile dementia was significantly increased; hyperhomocysteinemia could also directly cause cardiovascular damage and promote the occurrence of vascular remodeling. The results of the study by Temple et al. (67) showed that hyperhomocysteine was not only positively correlated with intracranial artery stenosis, but also had some relationship with coronary artery stenosis. Have reported that Hcy has an effect on CHD and is an independent risk factor for CHD that predicts the severity of CHD (68). Xiong et al. (69) found that with the increase of the number of coronary artery lesions and the degree of stenosis, Hcy levels gradually increased, which can be used to predict the degree of coronary artery stenosis.

### High lipoprotein (a) [LP (a)]

Lp (a) is actually a cholesterol-rich lipoprotein, whose structure is similar to that of low-density lipoprotein, which is composed of low-density lipoprotein and apolipoprotein (a), and is an independent lipoprotein. High plasma concentration of LP (a) can promote the occurrence of AS. At the same time, high LP (a) is also an important and independent risk factor for the occurrence of cardiovascular and cerebrovascular atherosclerosis, and it is also one of the genetic markers for early occurrence of coronary heart disease and cerebrovascular disease. The plasma concentration of LP (a) is mainly determined by genes, accounting for about 90%. Whether there is racial difference remains to be further studied.

At present, LP (a) is recognized as a risk factor for coronary heart disease and stroke. Studies have shown that there are lipoprotein (a) in serum and AS plaque, women are higher than men, plaque concentration is higher than serum, unstable plaque is higher than stable plaque (70). Some scholars (71) studied the relationship between LP (a) and IMT and found that LP (a) level was correlated with carotid IMT thickness, and the increase of LP (a) level was an independent risk factor for the aggravation of CAS. At the same time, LP (a) can also lead to coronary artery stenosis. Some clinical observations (72) suggest that serum Lp (a) content is closely related to the lesion range of coronary artery stenosis, and the number of diseased coronary arteries increases with the increase of Lp (a) content.

### History of smoking

The view that smoking is an independent risk factor for intracranial artery stenosis has also been fully confirmed. However, most researchers believe that smoking is an important influencing factor atherosclerosis. Related studies in China have shown that smoking is closely related to the occurrence of intracranial and extracranial atherosclerosis (73). Cheng et al. (74) showed that smoking was closely related to the aggravation of AS, which could increase the progression rate of AS by 50%, especially for patients with hypertension. Studies have also shown that the probability of AS in smokers and non-smokers is significantly different. After eliminating the influence of potential risk factors, smoking is closely related to the incidence of intracranial artery stenosis (75). Smoking is an important factor in coronary artery disease. It is generally believed that the influence of smoking on patients not only occurs in the cerebral vessels, but also the damage of smoking on patients can affect the whole blood vessels. A survey in the United States suggests that the death caused by smoking-related cardiovascular and cerebrovascular diseases accounts for 32%, even higher than that caused by smoking-related lung cancer (28%) (76). In patients with ischemic cerebral infarction, the greater the amount of smoking, the longer the time, the more serious the degree of intracranial artery disease. However, most smoking patients with cerebrovascular disease tend to ignore the progress of smoking on intracranial artery disease, and ultimately lead to the occurrence of cardiovascular and cerebrovascular diseases.

### Long-term history of alcoholism

Most studies have shown a J-shaped curve between alcohol intake and the risk of cerebrovascular disease, that is, mild drinking may have a protective effect on cerebrovascular disease, but moderate or even excessive drinking can lead to a sharp increase in the prevalence of cerebrovascular disease. Melgaard et al. (77) studied the local cerebral blood flow in imaging technology to study the local cerebral blood flow in alcohol abuse group and control group, and found that the cerebral blood flow in alcohol abuse group was significantly lower than that in that in the normal control group. Gdovinová (78) studied the blood flow of the middle cerebral artery in patients admitted to hospital due to long-term alcohol abuse and alcohol withdrawal symptoms. Compared with the control group, it was shown that heavy drinking could slow down the blood flow velocity of the middle cerebral artery.

### Obesity/overweight

Obesity or overweight has been paid more and more attention by cerebrovascular disease researchers. Zhang et al. (79) found that the incidence of cerebrovascular disease in middle-aged male patients with obesity was more than twice that of normal people. At the same time, it was also found that the incidence of cerebrovascular disease will increase by 18% when BMI increased by 2 units. Milionis et al. (80) believe that central obesity in patients with metabolic syndrome is associated with increased prevalence of cerebrovascular disease in patients over 70 years of age; Meta-analysis of the relationship between obesity and coronary heart disease by Song et al. (81) showed that BMI > 25 kg/m^2^ was a risk factor for coronary heart disease, and abdominal obesity became an important risk factor for coronary heart disease. Obesity or overweight may not lead to cardiovascular and cerebrovascular diseases through a single pathway. Studies have revealed that the mechanism by which increased BMI leads to arterial stenosis and cerebrovascular disease: the increase of BMI is closely related to cellular inflammatory response, abnormal blood lipid metabolism, increase of oxidative free radicals, increased platelet aggregation ability, increased coagulation function, and decreased activity of the system in the state of insulin resistance and insulin resistance, leading to atherosclerosis and plaque formation under the interaction of various factors (82).

### Hyperuricemia

Uric acid is the final product of human exogenous (diet) and endogenous purine metabolism, mainly in the liver and intestine through xanthine dehydrogenase and xanthine oxidase oxidation hypoxanthine, xanthine, uric acid. Under ischemia or hypoxia, xanthine dehydrogenase is reversible converted into xanthine oxidase, and the increase of uric acid substrate can produce a large amount of uric acid. Hyperuricemia (HUA) is one of the risk factors of intracranial artery stenosis. Zhang et al. (83) found that the content of serum uric acid was significantly increased in patients with severe intracranial artery stenosis, suggesting that the content of uric acid is closely related to the degree of intracranial artery disease. Kim et al. (84) confirmed that HUA is closely related to intracranial artery stenosis, that is, the incidence of intracranial artery stenosis in HUA patients is twice that of the normal group, indicating that uric acid levels indicate the severity of stenosis. HUA affects the prognosis of patients with ICAD. Early experiments abroad have confirmed that there is a high uric acid in the plaque of human atherosclerosis, indicating that uric acid has a direct effect on the formation of atherosclerosis. High uric acid levels not only cause intracranial artery stenosis, but also promote the occurrence of carotid artery and coronary artery stenosis. The famous JACD study in Japan (85) strongly confirmed the correlation between hyperuricemia (HUA) and coronary artery stenosis.

### High-sensitivity C-reactive protein

Serum high sensitivity C reactive protein (Hs-CRP) is a phase protein synthesized by the liver, which stimulates the complement system and leads to the occurrence of inflammatory response. It is an independent risk factor for cardiovascular disease (86). Hs-CRP0 has long half-life, high detection stability and little difference between day and night. It is not affected by food intake, age and gender. The increase of serum Hs-CRP0 not only has guiding significance for inflammatory response, but also promotes inflammatory response and plaque rupture. The level of Hs-CRP0 is significantly correlated with the stability of coronary plaque. Ding et al. (87) confirmed that the level of Hs-CRP in serum was related to the echo intensity and surface morphology of atherosclerotic plaques. At the same time, studies have shown (88) that the degree of coronary stenosis in CHD patients is significantly correlated with the level of Hs-CRP, which can be used to determine the degree of coronary lesions.

### Lifestyle behaviors

Lifestyle behaviors including diet, physical activity and sedentary behavior are key modifiable risk factors for many diseases and improving these behaviors is considered essential to reducing the financial and health burden of diseases. Unhealthy diet, physical inactivity and sedentary behavior are known to track from childhood into adulthood and are difficult to change later in life. This exacerbates associated health problems and demonstrates why preventing the development of these health risk factors throughout the lifespan is important. It is reported that dyslipidaemia accelerates the atherosclerotic process and its morbid consequences (89). The factors that are known to influence lipid levels and metabolism include dietary fat intake, alcohol intake, and levels and intensity of physical activity (90). According to the American Heart Association (91), atherosclerosis is a leading cause of cardiovascular disease. Known factors that contribute to the development of atherosclerosis include high low-density lipoprotein cholesterol (LDL-C), low high-density lipoprotein cholesterol (HDL-C), high triglycerides (TG), obesity, a poor diet, physical inactivity, hypertension, genetics, smoking, diabetes mellitus, and the environment. The study (92) found that the lower occurrence of cancer and cardiovascular disease in the population around the Mediterranean basin has been linked to the dietary habits of the region. Such a diet is rich in nuts, fruits, vegetables, legumes, whole-wheat bread, fish, and olive oil. Components of the Mediterranean diet are an important source of antioxidant and anti-inflammatory molecules, among which omega-3 fatty acids, oleic acid, and phenolic compounds are prominent. In addition, the study of Jeong et al. (93) showed that individuals with cardiovascular disease may benefit from physical activity to a greater extent than do healthy subjects without cardiovascular disease.

With the development of science and technology, the research on the correlation between intracranial artery, carotid artery and coronary artery stenosis is deepening. Whether there is a correlation between them is still controversial. Most studies believe that there is a correlation between them, and some scholars believe that there is no correlation between them. However, we find that with the aggravation of the scope and degree of coronary artery disease, the degree of intracranial and extracranial artery stenosis is also increasing. At the same time, it is also recognized that a variety of risk factors increase the degree of intracranial, carotid and coronary artery stenosis at the same time, but the impact of various risk factors on the degree of intracranial and extracranial artery and coronary artery stenosis is different. In addition, some patients have ischemic stroke first, and then coronary heart disease. Some patients have coronary heart disease first, and then ischemic cerebrovascular disease, which may be affected by genetic, environmental and other risk factors. As for the specific reasons, it is still unclear, and further research is needed. Although there is a certain relationship between intracranial artery stenosis, carotid artery stenosis and coronary artery stenosis in ICAD patients, there is no consensus on whether it is necessary to perform routine coronary CTA screening for ICAD in patients with intracranial and extracranial artery stenosis. Whether the ICAD patients with intracranial and extracranial artery stenosis and coronary cloud vein stenosis are given conventional drug therapy for coronary heart disease is still not conclusive. Further multi-center, large sample and prospective studies are still needed to guide clinicians in the prevention, treatment, condition judgment or prognosis of cardiovascular and cerebrovascular diseases.

## Author contributions

RL and JS conceived of the study. JS participated in its design and coordination. JS and RL helped to draft the manuscript. All authors read and approved the final manuscript.

## Conflict of interest

The authors declare that the research was conducted in the absence of any commercial or financial relationships that could be construed as a potential conflict of interest.

## Publisher's note

All claims expressed in this article are solely those of the authors and do not necessarily represent those of their affiliated organizations, or those of the publisher, the editors and the reviewers. Any product that may be evaluated in this article, or claim that may be made by its manufacturer, is not guaranteed or endorsed by the publisher.
